# Use of Project ECHO in Response to COVID-19 in Countries Supported by US President’s Emergency Plan for AIDS Relief

**DOI:** 10.3201/eid2813.220165

**Published:** 2022-12

**Authors:** Janell Wright, Laura Tison, Helen Chun, Cristine Gutierrez, Mariangeli Freitas Ning, Rosa Elena Morales, Beatriz Lopez, James Simpungwe, Kenneth Masamaro, Nazira Usmanova, Gram Mutandi, Sudhir Bunga, Simon Agolory

**Affiliations:** US Centers for Disease Control and Prevention, Guatemala City, Guatemala (J. Wright, C. Gutierrez, M. Freitas Ning, R.E. Morales, B. Lopez);; US Centers for Disease Control and Prevention, Atlanta, Georgia, USA (L. Tison, H. Chun);; US Centers for Disease Control and Prevention, Lusaka, Zambia (J. Simpungwe, S. Agolory);; US Centers for Disease Control and Prevention, Nairobi, Kenya (K. Masamaro);; US Centers for Disease Control and Prevention, Bishkek, Kyrgyzstan (N. Usmanova);; US Centers for Disease Control and Prevention, Windhoek, Namibia (G. Mutandi);; US Centers for Disease Control and Prevention, Juba, South Sudan (S. Bunga)

**Keywords:** Project ECHO, COVID-19, telementoring, HIV/AIDS, coronavirus disease, SARS-CoV-2, severe acute respiratory syndrome coronavirus 2, viruses, respiratory infections, zoonoses, US President’s Emergency Plan for AIDS Relief, South Sudan, Namibia, Zambia, Kyrgyz Republic, United States

## Abstract

The US Centers for Disease Control and Prevention, with funding from the US President’s Plan for Emergency Relief, implements a virtual model for clinical mentorship, Project Extension for Community Healthcare Outcomes (ECHO), worldwide to connect multidisciplinary teams of healthcare workers (HCWs) with specialists to build capacity to respond to the HIV epidemic. The emergence of and quick evolution of the COVID-19 pandemic created the need and opportunity for the use of the Project ECHO model to help address the knowledge requirements of HCW responding to COVID-19 while maintaining HCW safety through social distancing. We describe the implementation experiences of Project ECHO in 5 Centers for Disease Control and Prevention programs as part of their COVID-19 response, in which existing platforms were used to rapidly disseminate relevant, up-to-date COVID-19–related clinical information to a large, multidisciplinary audience of stakeholders within their healthcare systems.

The onset of the COVID-19 pandemic challenged health systems worldwide, resulting in service delivery disruptions and compromised quality of care of illnesses worldwide. HIV services were no exception to this phenomenon; continuity of HIV prevention and treatment was severely affected (*1*). Approximate excess deaths caused by HIV and AIDS of >400,000 persons in 2020 has been estimated as a result of COVID-19–induced disruptions (*1*). COVID-19 has affected the global response to HIV and AIDS, and countries that implemented adaptive mitigation measures for health services’ continuity have reported fewer negative effects than countries that did not (*2*).

Project Extension for Community Healthcare Outcomes (Project ECHO) was launched in 2003 by the University of New Mexico Health Sciences Center (Albuquerque, NM, USA) to expand access to hepatitis C treatment for patients living in remote areas of the state. Through a hub-and-spoke model that connects spoke sites to a centrally located hub of subject matter experts through video conferencing technology, Project ECHO uses case-based learning to build communities of practice and learning among geographically distant providers practicing at different levels of the healthcare system (*3*). Since its inception in 2003, the ECHO model has been adapted to address a variety of healthcare workforce development needs and expanded to multiple geographic locations (*4*). As one of the first countries in Africa to adopt Project ECHO, Namibia connected remote clinical sites with centrally located specialists for HIV and tuberculosis (TB) medical education and care management in 2015. All major district hospitals and high-volume healthcare centers in the country are now connected by this platform.

The COVID-19 pandemic has posed numerous unforeseen challenges to HIV service delivery Programs and sites supported by the US President’s Plan for Emergency Relief (PEPFAR) have faced the need to develop and adapt creative solutions for ongoing frontline provider support and HIV service quality assurance in this context. Traditional in-person training and site visit approaches were no longer feasible or recommended because of restrictions on in-person gatherings and the priority of preserving the safety of providers and beneficiaries and limiting COVID-19 spread. We describe national and regional examples of how the Project ECHO platform was used to build capacity, rapidly and regularly disseminate evolving information on COVID-19 prevention and treatment in people living with HIV, and mentor frontline providers in resource-poor health settings supported by PEPFAR.

## Methods

Respondents from a convenience sample of 9 PEPFAR-supported countries known to have implemented Project ECHO for their HIV and TB programs before the COVID-19 pandemic completed a template to capture whether and how Project ECHO was being used for COVID-19–related topics, session frequency, number of participants, cadre type, and geographic location. The study team entered the data into a Microsoft Excel (https://www.microsoft.com) spreadsheet for data organization and descriptive analyses. Respondents implementing COVID-19 Project ECHO sessions answered a separate open-ended questionnaire about implementation challenges, program facilitators, and lessons learned from the use of the ECHO model to address COVID-19 (Appendix). The study team entered responses in Microsoft Word and grouped common themes related to implementation-enabling factors and challenges and perceived public health benefits. This project was reviewed in accordance with US Centers for Disease Control and Prevention (CDC) human research protection procedures and was determined to be nonresearch.

### HIV Project ECHO Programs Incorporating COVID-19 Topics

In 4 countries and 1 region (South Sudan, Namibia, Zambia, Kyrgyz Republic, and Central America), existing HIV Project ECHO programs were used to incorporate COVID-19 topics (Table 1). Although most of these programs targeted doctors and nurses, some also included other healthcare workers. For example, in South Sudan, ECHO sessions included participants from multiple cadres, such as clinical monitoring and evaluation staff, psychosocial counselors, laboratorians, and community health workers. Namibia included laboratorians; Zambia, pharmacists; and Kyrgyz Republic, general practitioners. Project ECHO sessions began including COVID-19 topics between January and December 2020; most started in March, around the time countries and regions began to report COVID-19 cases.

**Table 1:** Characteristics of projects Incorporating COVID-19 topics in HIV/TB Project ECHO programs*

Project ECHO characteristics	Coordinating organizations	COVID-19 topics covered during ECHO sessions	Main COVID-19 topics
South Sudan HIV ECHO: first session Mar 11, 2020, and occurred weekly; range 219–322 participants.	ICAP South Sudan, College of Physicians and Surgeons of South Sudan, Juba Teaching Hospital (Central Equatoria State)	1) Introduction to COVID-19; 2) protecting frontline healthcare workers to ensure continuity of services; 3) case management 1: mild/moderate and severe cases; 4) case management 2: critical cases and special populations; 5) infection prevention and control; 6) patient screening, triage, isolation, and contact tracing; 7) rational use of PPE; 8) cleaning and waste/dead body management	1) Case management 1: mild/ moderate and severe cases; 2) case management 2: critical cases and special populations; 3) infection prevention and control
Namibia ECHO: first session Mar 17, 2020, and occurred weekly; range 172–390 participants	Namibia Ministry of Health and Social Services	1) COVID-19 and patients on ART; 2) HIV patient management in the COVID-19 context; 3) overview of infection prevention and control measures in COVID-19 pandemic; 4) national update on COVID-19 developments; 5) pediatric HIV disclosure in the context of COVID-19; 6) how to prepare ART clinics for COVID-19	1) Case management 1: basics of COVID-19, management of mild/moderate and severe cases 2) case management 2: advanced management of critical cases and special populations; 3) infection prevention and control; 4) Introduction and planning for IPC: WHO Tabletop Exercise
Zambia Project ECHO TB/HIV: first session Jan 29, 2020, and occurred weekly with ad hoc sessions; range 64–65 participants	Zambia Ministry of Health	1) Clinical update on the Novel Coronavirus (2019-nCoV); 2) COVID-19 orientation for HCWs; 3) COVID-19 pandemic response; 4) COVID-19 in children; 5) PPE donning and doffing; 6) clinical features of COVID-19; 7) COVID-19 pandemic literature review; 8) psychological aspects of the COVID-19 pandemic; 9) COVID-19 management – experiences from China and Italy; 10) COVID-19 and management of noncommunicable diseases and comorbidities; 11) ensuring quality HIV services during COVID-19 pandemic; 12) COVID-19 in Zambia: “What We Should Know”; 13) TB/TPT guidance during the COVID-19 pandemic; 14) COVID-19 vaccine	1) Sustaining quality HIV services amidst COVID-19; 2) clinical features of COVID-19; 3) COVID-19 vaccination
Kyrgyzstan HIV ECHO Project: first session Sep 23, 2016, and occurred biweekly and weekly; range 30–60 participants.	Kyrgyz State Medical Institute for Postgraduate Education	Introduction to COVID-19	1) Outpatient COVID-19 management; 2) COVID-19 diagnosis, clinical features, and management; 3) etiology, clinical features; 4) diagnostics and treatment
Central America HIV Treatment ECHO: first session Oct 9, 2020, and occurred weekly; range 31–87 participants.	SE-COMISCA El Salvador	COVID-19 and HIV co-infection	COVID-19 and HIV co-infection

*ART, antiretroviral therapy; ECHO, Extension for Community Healthcare Outcomes; IPC, infection prevention and control; PPE, personal protective equipment; SE-COMISCA, Secretaría Ejecutiva del Consejo de Ministros de Salud de Centroamérica y República Dominicana; TB, tuberculosis; TPT, tuberculosis preventive treatment; WHO, World Health Organization.

### COVID-19–Focused Project ECHO Programs

In total, 4 ECHO programs (2 in Central America and 1 each in Kenya and Southern Africa) were focused on COVID-19–related content (Table 2). COVID-19 Project ECHO programs in Central America addressed laboratory-specific and clinical-specific topics. Of those 4 programs, 3 catered to audiences within the broader geographic region (2 in Central America and 1 in southern Africa). ECHO session frequency varied from weekly in the Central America COVID-19 Clinical ECHO program to biweekly for the COVID-19 Laboratory ECHO program in Central America and southern Africa and monthly for the Kenya national COVID-19 ECHO program. Similar to the HIV Project ECHO programs that incorporated COVID-19 topics, almost all programs that were COVID-19–focused included multidisciplinary participants (physicians, nurses, clinical officers, pharmacists, and laboratory staff); the exception was the Central America COVID-19 laboratory ECHO program, which only targeted laboratorians. 

**Table 2:** COVID-19 Project ECHO program characteristics from 4 regions and countries, 2020–2021*

Project ECHO COVID-19 characteristics	Coordinating organization (Hub)	No. participants	Participant cadre	Participant location
Regional Central America Laboratory COVID-19 Project ECHO: in Spanish; first session held Jun 9, 2020; 6 biweekly sessions.	SE-COMISCA El Salvador	101–224	Laboratory staff	Member states of the SICA region (Belize, Costa Rica, Guatemala, El Salvador, Honduras, Nicaragua, Panama, Dominican Republic), Bolivia, Ecuador, Mexico, Peru, United States
Regional Central America Clinical COVID-19 Project ECHO: in Spanish; first session held Apr 15, 2020; 31 weekly sessions.	SE-COMISCA El Salvador	127–328	Medical doctors, nurses, clinical officers/medical licentiates, pharmacists	Member states of the SICA region (Belize, Costa Rica, Guatemala, El Salvador, Honduras, Nicaragua, Panama, Dominican Republic), Bolivia, Ecuador, Mexico, Peru, Spain, United States
Kenya COVID-19 Project ECHO: in English; first session held Apr 3, 2020; 5 monthly sessions.	National AIDS and STI Control Council Hub, Kenyatta National Hospital Hub	300–1,037	Physicians, medical officers, clinical officers, nurses, pharmacists, pharmaceutical technologists, laboratory staff, infection prevention teams	Kenya
Southern Africa Regional COVID-19 Project ECHO (SARE): in English with Portuguese translation; first session held Dec 3, 2020; 11 biweekly sessions.	Zambia Ministry of Health Project ECHO	61–264	Medical doctors, nurses, clinical officers/medical licentiates, pharmacists	Botswana, Eswatini, Lesotho, Malawi, Mozambique, Zambia

*ECHO, Extension for Community Healthcare Outcomes; SE-COMISCA, Secretaría Ejecutiva del Consejo de Ministros de Salud de Centroamérica y República Dominicana; SICA, Sistema de la Integración Centroamericana; STI, sexually transmitted infection.

## Results

### Enabling Factors for Implementation

Country programs using Project ECHO during the COVID-19 pandemic cited several key enabling factors for implementation. Three of four COVID-19–focused Project ECHO programs launched from existing national ECHO hubs; in doing so, those programs capitalized on previously established information technology networks, equipment, and staff knowledge of ECHO. The Central America CDC program had an existing partnership with the regional ECHO hub that hosts both the TB- and HIV-focused Project ECHO programs, which provided a foundation to rapidly launch the COVID-19–focused ECHO program. Through its established network of ECHO participants, the Central America clinical COVID-19 ECHO program quickly connected to almost 4,000 healthcare providers who had participated in HIV- and TB-focused ECHO sessions over the previous year. This immediate network enabled rapid and broad dissemination of evolving COVID-19 diagnosis and management information. Similarly, the South Sudan HIV Project ECHO hub, established in 2018, built on its existing network to incorporate COVID-19 topics into their existing HIV Project ECHO program and expanded their reach to medical teams in 40 health facilities. Zambia respondents cited the ECHO hub location within the national Ministry of Health and its connection with 10 provincial health offices throughout the country as a key enabling factor in reaching healthcare providers across the country. In Central America, support from the Executive Secretary of the Regional Ministries of Health partner (SE-COMISCA) was critical to establish regional support for Project ECHO. The CDC Central America COVID-19 Project ECHO noted that its previous experience drawing on the expertise of diverse local and national health experts from the Pan American Health Organization, ministries of health, and large hospitals, as well as local healthcare personnel, for planning, facilitation, and capacity building contributed to high attendance and reported satisfaction with sessions, which was assessed through anonymous polling at the end of sessions.

Other factors identified by country teams that aided in implementation included the virtual delivery method and reasonable time requirements of Project ECHO. Respondents in Zambia described the weekly, 60-to-90–minute format of Project ECHO sessions as “ideal to minimize disruptions in clinical duties” and noted “the flexibility of tailoring ECHO sessions to meet the specific healthcare worker COVID-19 topic needs as opposed to strict adherence to a predetermined curriculum.”

### Public Health Benefit

Respondents described the perceived public health benefits of using Project ECHO to respond to COVID-19. One common theme emerged regarding the benefit of bidirectional information sharing between geographically distant frontline providers and health system leaders, which helped provide insight into the public health policy and broader service delivery challenges and ability to disseminate evolving guidelines and policies for more rapid adoption. Respondents from the Project ECHO Laboratory program in Central America indicated question-and-answer sessions were helpful in fostering dialogue between facility-level laboratory staff and national-level persons who might be responsible for influencing COVID-19 laboratory policies and procedures. The South Sudan respondents highlighted how including COVID-19 topics in their HIV Project ECHO program was “crucial to information dissemination in an extremely challenging operating environment where public health programs and impact otherwise suffer from poor physical access, limited human resource capacity, insecurity and limited-service quality oversight and supervision.”

### Challenges to Implementation

Countries noted several challenges to implementing Project ECHO during and with COVID-19. Those included lack of time to identify the quantity and quality of experts who were needed to present or assist with sessions, the large volume of rapidly evolving and often difficult-to-navigate information on COVID-19 prevention and clinical management (Table 3), limited ability to maintain interactive discussion-oriented sessions while disseminating large quantities of information within the allocated time, and difficulty with long-term session planning.

**Table 3:** Topics, gaps and participant concerns and questions in COVID-19 Project ECHO sessions conducted in countries supported by US President’s Emergency Plan for AIDS Relief, 2020–2021*

Topics covered	Gaps identified by participants	Examples of main concerns and common questions
COVID-19 case management	1) Principles of oxygen escalation/de-escalation; 2) innovative therapies for mild/moderate cases	1) What parameters are used in the decision to use supportive oxygen?; 2) mild/moderate COVID-19 case management; 3) how long does immunity to COVID-19 last?
Co-infection and comorbidities	1) Warning signs and management of cardiovascular manifestations of COVID-19; 2) management of patients with hypertension and COVID-19; 3) COVID-19 management in patients with comorbidities	1) How to standardize treatment for patients; 2) the role of steroid management in COVID-19 management
Infection prevention and control, PPE	1) PPE principles and use/reuse scenarios; 2) infection prevention and control in the context of community service delivery; 3) SARS-CoV-2 modes of transmission	1) Principles of donning and doffing PPE for frontline HCWs and standards for reuse in resource-limited settings; 2) mask use according to clinical service delivery points; 3) community behavior change strategies in infection prevention
Vaccines/immunization	1) Vaccine development processes and mechanisms of action; 2) COVID-19 vaccine demand creation strategies	1) Vaccination guidance for pregnant patients, other vulnerable populations, and persons previously infected with COVID-19; 2) management and reporting of vaccine-related adverse events during vaccination campaigns
Mental health	1) Specialists available and equipped to address HCW needs; 2) strategies to address mental health issues and build resilience among front-line HCW	1) Addressing mental health needs of HCWs during the pandemic; 2) HCW support systems and lack thereof; 3) insufficient expertise in diagnosing Mental Health issues leading to a growing number of undiagnosed health issues
Surveillance	1) SARS-CoV-2 surveillance methods and best practices; 2) acute febrile disease surveillance in a COVID-19 pandemic	1) Role of community health workers for COVID-19 disease surveillance; 2) harnessing digital technologies to improve Health Information systems
Laboratory	1) Role of public health laboratories in the pandemic response; 2) SARS-CoV-2 detection kit evaluation and validation; 3) SARS-CoV-2 diagnostic testing expansion and decentralization	1) Diagnostic test result (molecular and antigen) interpretation; 2) COVID-19 test positivity and duration of infectivity?; 3) serologic tests’ utility in the diagnosis of acute COVID-19

*ECHO, Extension for Community Healthcare Outcomes; HCW, healthcare worker; PPE, personal protective equipment.

Country and regional programs reported variable participation in Project ECHO sessions. In addition, CDC country staff noted information technology connectivity challenges and session-timing conflicts with clinical duties as barriers to consistent participation.

### Limitations

One of the limitations of this analysis is the lack of a systematic review of all Project ECHO programs globally that were implemented in response to the COVID-19 pandemic. We used a convenience sample, limiting the generalizability of observations or conclusions beyond the contributing countries. The tool to capture Project ECHO program characteristics for this analysis was limited, and a more in-depth comprehensive tool to systematically evaluate Project ECHO programs during COVID-19 is likely needed. In addition, observing the development of communities of practice, a core function of any ECHO program, might have been limited by variable participation across ECHO programs.

## Conclusions

The COVID-19 pandemic heightened existing concerns over disruptions in healthcare service delivery and essential public health functions during public health emergencies. Project ECHO might help address some of these concerns by enabling the consistent delivery of clinical and public health updates and engaging communities of providers. The ability to connect multiple stakeholders could help strengthen service quality and system resilience in the face of new challenges such as COVID-19 and lead to potential long-term positive outcomes. Evaluating ECHO programs formally to establish implementation best practices and recommendations for the use of this platform could benefit the larger public health community in its response to future public health threats.

Appendix Additional information about use of Project ECHO in response to COVID-19 in countries supported by President’s Emergency Plan for AIDS Relief, 2020–2021
