# Supplementary material for: Use of Project ECHO in Response to COVID-19 in Countries Supported by US President’s Emergency Plan for AIDS Relief
Source: Emerg Infect Dis. 2022 Dec;28(Suppl 1):S191–6. doi: 10.3201/eid2813.220165 (PMC9745236; doi:10.3201/eid2813.220165)
Supplement: Appendix — Additional information about use of Project ECHO in response to COVID-19 in countries supported by President’s Emergency Plan for AIDS Relief, 2020–2021 [file 22-0165-Techapp-s1.pdf]

Article DOI: https://doi.org/10.3201/eid2813.220165

Use of Project ECHO in Response to COVID-19 in Countries Supported by US President's Emergency Plan for AIDS Relief

Appendix

A. Questionnaire provided to contributing authors on Project ECHO experiences during COVID-19 pandemic response

Project ECHO COVID-19 Lessons Learned

Instructions: Please answer the questions with as much description as possible. Limit the final document to 2 pages. Send to Janell Wright hxx3@cdc.gov and Cristine Gutierrez qgq6@cdc.gov by June 2 by 5 EST. (Note- submissions after this may not be considered in the final manuscript)

Author/s: (max 2 per program and from CDC only)

1. What were the top 3 challenges in implementing COVID-19 Project ECHO?

2. What were the top 3 main drivers and facilitators to the launch of COVID-19 Project ECHO?

3. How did you decide to use Project ECHO over other virtual training modalities (webinars, etc.)?

4. What were the top 3 lessons learned implementing a virtual community of practice through Project ECHO COVID-19?

5. How did the Project ECHO COVID-19 contribute to the response to COVID-19 in your country or region?

6. What is the future of Project ECHO in your country/region?

A. Excel table templates for Project ECHO details sent via email to collaborating authors

Template: Project ECHO with COVID-19 Topics

Name of Project ECHO TB/HIV/HEP C/ other (including COVID-19 topics)	Hub name _ org affiliation_location	Language	Target audience by profession	Date of first session	Frequency	No. participants	Attendance range	Mean attendance per session	Licensures or titles of participants	Locations of participants for all sessions	Sessions in the last year that covered COVID-19 topics (didactic)	Sessions in the last year that covered COVID-19 topics (case presentations)	Top 3 topics covering COVID-19	Information submitted by (name and username)

Template: COVID-19 Project ECHO Sessions

Name of Project ECHO COVID-19	Topics	No. sessions on the topic	No. participants per session	Identified gaps	Top 3 asked questions or concerns	% of time spent per session		
						Didactic presentations	Cases presentations	Q&A

Template: COVID-19 Project ECHO

Name of Project ECHO COVID-19	Date of first confirmed case of COVID-19 in country or region	Date of first session	Level (city, state/province, national, regional/many countries)	Hub name _ org affiliation_location	Language	Target audience by profession	No. sessions	Frequency (weekly, biweekly, monthly)	No. participants	Attendance range	Mean attendance per session	% of participants attending 50% of total sessions or sessions to date	Licensures or titles of participants	Locations of participants for all sessions (example: Regional (Guatemala, Panama and El Salvador) or Guatemala (Guatemala City, Zacapa and Flores)	Information submitted by (name and username)
